# Replacing nitrogen fertilizer with organic alternatives changes the soil microbial community diversity in Northwest China’s arid farmland, leading to increased potato yields

**DOI:** 10.3389/fpls.2026.1846452

**Published:** 2026-06-17

**Authors:** Xiaohua Shi, Yuanyuan Zhang, Binhao Xu, Yonglin Qin, Yang Chen, Jing Yu, Kun Liu, Lan Wu, Junmei Liang, Zhi Jin, Mingshou Fan, Liguo Jia

**Affiliations:** 1 Inner Mongolia Agricultural University, Hohhot, China; 2 Inner Mongolia Academy of Agricultural & Animal Husbandry Sciences, Hohhot, China; 3 Ulanqab Agricultural & Animal Husbandry Ecological Resource Protection Center, Jining, China

**Keywords:** microorganism, nitrogen fertilizer substitution, organic fertilizer, potato, rhizosphere soil

## Abstract

Northwest China, a major potato-producing region, relies heavily on chemical nitrogen fertilizers to maintain yields. In this arid area with limited rainfall and low soil organic matter, however, excessive nitrogen use impairs soil structure and disturbs nutrient balance, ultimately degrading soil quality and undermining sustainable agriculture. Therefore, understanding whether organic fertilizers can replace chemical nitrogen to improve soil quality and stabilize yields is essential. In this study, we applied six fertilization treatments and used 16S rRNA and ITS high-throughput sequencing to analyze potato rhizosphere microbial communities. We found that replacing 60% of chemical nitrogen with organic fertilizer was the optimal strategy for potato production in this arid region; this substitution increased potato yields by 8.23% compared with chemical fertilizer alone. This substitution also significantly reduced soil bulk density, enhanced the formation and stability of soil water-stable aggregates, and increased the levels of total nitrogen (TN), soil organic carbon (SOC), ammonium (NH_4+_-N), and nitrate (NO_3-_-N). Organic fertilizer had a more pronounced effect on bacterial diversity than fungal diversity, notably boosting the populations of Proteobacteria and Actinobacteria. The fungal community was primarily composed of Ascomycota and Basidiomycota. Key factors influencing bacterial community changes included SOC, NH_4+_-N, NO_3-_-N, and pH, while SOC and the carbon-to-nitrogen (C:N) ratio mainly affected the fungal community. In summary, substituting nitrogen fertilizers with organic alternatives can enhance soil physical and chemical properties, boost potato yields, and significantly alter the rhizosphere microbial community structure. This provides a scientific foundation for mitigating soil quality degradation in northern China’s arid regions and promoting sustainable and efficient fertilization practices.^1^

## 1 Introduction

Potato (*Solanum tuberosum L.*) ranks among the world’s vital food and economic crops, with its cultivation area and total output following rice, wheat, and corn. It plays a crucial role in ensuring food security and promoting sustainable agriculture (Atapattu et al., 2025). The arid region of Northwest China, characterized by an arid climate, uneven precipitation distribution, and low soil organic matter, is a significant potato-producing area. Long-term intensive cultivation and reliance on chemical fertilizers have led to soil quality degradation in this region, characterized by organic matter depletion, poor soil structure, and reduced nutrient cycling efficiency. In North China’s arid areas, the soil’s weak buffering capacity and limited carbon pool, coupled with water-restricted microbial activity, make it more susceptible to quality decline from sole chemical fertilizer use (Wakgari, 2021). In this region, long-term intensive farming and heavy reliance on chemical fertilizers have led to soil quality degradation. Common issues include organic matter depletion, poor soil structure, and reduced nutrient cycling efficiency. However, potato production here heavily relies on chemical fertilizers. The prolonged excessive use of nitrogen fertilizers, while maintaining crop yields, has led to ecological and environmental issues, especially in sandy soils, such as nitrogen leaching, increased greenhouse gas emissions, and soil degradation (Zhao et al., 2021; Chen et al., 2024b). Additionally, excessive nitrogen disrupts the soil microbial community, impairs soil ecosystem functions, and ultimately hinders the continuous improvement of potato yield and quality.

Partial replacement of chemical nitrogen fertilizers with organic fertilizers is a key strategy for achieving both high crop yields and environmental sustainability. In the arid regions of northern China, the soil exhibits a weak buffering capacity and a limited carbon pool. Additionally, microbial activity is often constrained by water scarcity. Relying solely on chemical fertilizers can lead to a decline in soil quality (Iqbal et al., 2022). Thus, supplying organic materials is essential to maintaining soil health and productivity. Organic fertilizers provide not only nitrogen, phosphorus, potassium, and various trace elements (Zhang et al., 2011) but also serve as a significant source of organic carbon. This carbon promotes soil aggregate formation and boosts soil organic matter. The nutrients in organic fertilizers are released gradually through microbial mineralization, which helps align nutrient supply with crop demand, reduces nutrient leaching risks, and enhances fertilizer efficiency (Shaji et al., 2021; Udumann et al., 2025). Research indicates that substituting a portion of nitrogen fertilizers with organic fertilizers can significantly boost crop yields and improve soil fertility compared to using chemical fertilizers alone (Hou et al., 2024). However, the yield benefits of organic fertilizers extend beyond direct nutrient supply, potentially involving complex soil biological processes.

Soil microorganisms are essential for regulating nutrient cycling and maintaining ecosystem stability. They also play a crucial role in linking fertilization practices to crop yield responses (Atapattu et al., 2025). The long-term use of chemical fertilizers alone often reduces microbial diversity and simplifies community structures, thereby inhibiting key functional microorganisms (Castiglione et al., 2021). Conversely, organic fertilizers can boost microbial metabolic activity, enhance carbon sequestration, and enrich beneficial microbial communities by providing both easily and slowly decomposable organic carbon substrates (Zhang et al., 2023). Research indicates that replacing 30%–70% of chemical nitrogen fertilizers with organic fertilizers significantly increases functional groups involved in organic matter decomposition and nitrogen transformation, such as actinomycetes and nitrogen-fixing bacteria. This shift also reduces the relative abundance of potential pathogens (Chen et al., 2024b; Wang et al., 2024), fostering a stable and efficient rhizosphere microbial system.

In recent years, high-throughput sequencing technology has significantly advanced soil microbial ecology research. This technology allows for detailed analysis of how fertilization affects microbial community structure and diversity (Lahlali et al., 2021). Studies have demonstrated that substituting nitrogen fertilizers with organic alternatives can notably change the β-diversity of soil microbial communities and reshape the microbial co-occurrence network (Han et al., 2025). For instance, Abudurezike, A. et al (Abudurezike et al., 2025)observed in a long-term field experiment in Xinjiang that increasing the proportion of organic nitrogen led to a significant enrichment of microbial taxa like Actinobacteria and Acidobacteria, known for decomposing complex organic matter. Conversely, the proportion of Proteobacteria, which thrive in nutrient-rich environments, decreased. This shift in community structure is often linked to soil organic matter content and nutrient slow-release properties, suggesting that organic fertilizers may enhance the rhizosphere micro-ecological environment by modulating microbial community functions, thereby promoting crop growth.

While previous studies have shed light on how substituting organic fertilizers for nitrogen fertilizers enhances soil ecological processes, there remains a gap in understanding within the potato production systems of Northwest China’s arid regions. Specifically, the mechanisms by which different ratios of organic fertilizer substitution influence the diversity and structural stability of the rhizosphere soil microbial community are not well-defined. Furthermore, the interaction between these microbial changes and soil physical and chemical properties, which ultimately affects potato yield, requires further exploration. Amid ongoing regional soil quality decline and the pressing need for yield stability, understanding microbial ecological mechanisms is crucial. This is particularly crucial for potatoes, a crop with high nutrient demands, where the pathways through which microorganisms contribute to yield increases have yet to be fully clarified.

This study focused on potatoes in the arid region of Northwest China. It systematically compared the effects of varying proportions of organic fertilizer replacing nitrogen fertilizer on potato yield and its components, soil physical and chemical properties, and the diversity and structure of bacterial and fungal communities in the rhizosphere soil. Specifically, we identified that 60% organic fertilizer substitution represents an optimal threshold that balances nutrient supply with microbial community enhancement, providing a quantitative reference for precision fertilization in arid agricultural systems. Furthermore, this study elucidates the differential responses of bacterial versus fungal communities to organic substitution, revealing that bacteria are more sensitive indicators of fertilization-induced soil quality changes. The aim was to uncover the mechanism by which organic fertilizer enhances potato yield from a microbial ecology perspective, providing a theoretical foundation for green and efficient potato fertilization and sustainable agricultural development in this region.

## 2 Materials and methods

### 2.1 General situation of the experimental area

**Figure 1:**
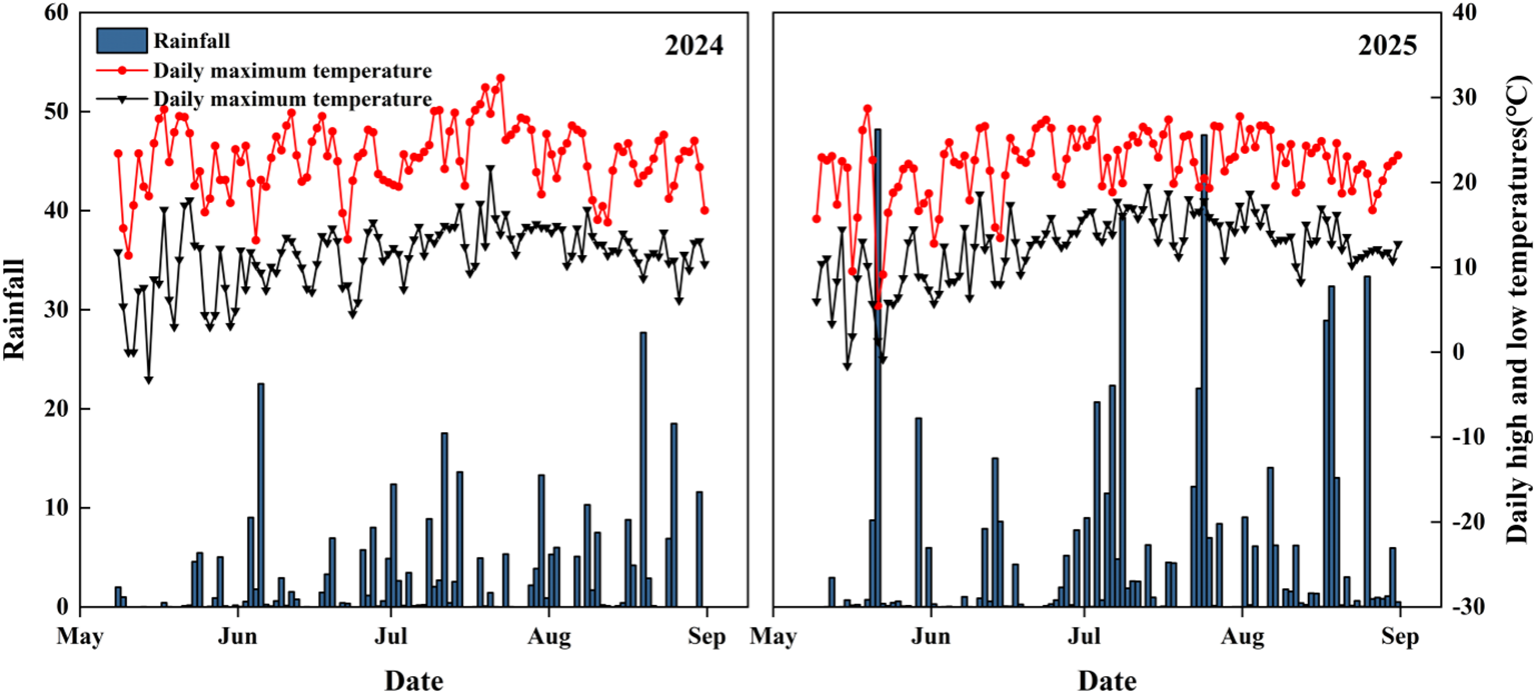
Daily temperature and precipitation during the potato growing season at the experimental site (Ulanqab, Inner Mongolia) in 2024 and 2025.

The experiment was conducted in Kebuer Town, Chahar Right Middle Banner, Ulanqab City, Inner Mongolia Autonomous Region (41°31′N, 112°65′E) over two consecutive growing seasons in 2024 and 2025. This area has a temperate continental climate, is located at an altitude of 1780 meters, and experiences an average annual temperature of 1.3 °C with about 542 mm of rainfall. According to the World Reference Base for Soil Resources (WRB) classification, The soil type is classified as chestnut soil, with a sandy loam texture (Guo et al., 2025). The average temperature, precipitation during the potato growing seasons in 2024 and 2025 are shown in **Figure 1**.In 2025, the initial physical and chemical properties of the 0–20 cm soil layer were documented as follows: soil organic carbon (SOC) was 12.87 g/kg, total nitrogen (TN) was 1.51 g/kg, available phosphorus (AP) was 14.3 mg/kg, available potassium (AK) was 128.5 mg/kg, and the pH was 8.12.

### 2.2 Test materials

The potato variety used in the experiment was Jinshu 16, which is widely cultivated in Inner Mongolia, has high yield potential, and belongs to the drought-resistant varieties. This variety is the main commercial cultivation variety in the arid potato-producing areas of northwest China (Gao, 2025). The commercial organic fertilizer employed was decomposed sheep manure, Manure is fully composted for 6 months to reach a mature state. containing 2.14% pure nitrogen (N), 1.24% phosphorus pentoxide (P_2_O_5_), and 2.18% potassium oxide (K_2_O). The chemical fertilizers tested included urea with 46% nitrogen (N), triple superphosphate with 46% phosphorus pentoxide (P_2_O_5_), and potassium sulfate with 51% potassium oxide (K_2_O).

### 2.3 Experimental design

The experiment employed a Completely Randomized Design (CRD) with six treatments and four replications. The six treatments were: no fertilization (CK), 100% chemical fertilizers (T0), organic fertilizer replacing 30% of nitrogen fertilizer (T1), organic fertilizer replacing 60% of nitrogen fertilizer (T2), organic fertilizer replacing 100% of nitrogen fertilizer (T3), and no application of nitrogen or organic fertilizer (N0). Each treatment was replicated four times. Each experimental plot measured 222.52 square meters (8.83 m wide × 25.2 m long). The recommended fertilizer dosage is based on the agricultural extension recommendations for potato production in Inner Mongolia, combined with conclusions drawn from multiple soil testing and formula experiments (Zhao, 2025). The replacement ratios for all treatments were based on equal nitrogen content, with N at 300 kg/hm², P_2_O_5_ at 210 kg/hm², and K_2_O at 315 kg/hm². All fertilizers, including organic, nitrogen, phosphorus, and potassium, were applied as basal fertilizers in a single application. The experiment was sown on May 9, 2024, and harvested on September 14, 2024; it was sown again on May 12, 2025, and harvested on September 16, 2025. The fertilization amounts for each treatment are detailed in **Table 1**.

**Table 1:** Fertilizer application rates of each treatment in the potato field experiment.

Treatment	Organic fertilizer (kg/hm^2^)	Fertilizer (kg/hm^2^)		
		N	P_2_O_5_	K_2_O
CK	0	0	0	0
T0	0	300	215	315
T1	4000	210	215	315
T2	8000	120	215	315
T3	14000	0	215	315
N0	0	0	215	315

### 2.4 Determination method for plant yield

During the 2024 and 2025 potato harvests, four 2 m² subplots were randomly selected from each experimental plot to evaluate tuber yield. Tubers were classified as large or small, using 150g as the threshold. Yield indicators, including the commercial potato rate, were then calculated. The calculation formula for the commodity potato rate is as follows:


$$\text{Commercial potato rate }(\%)=\;(\frac{\text{Weight of tubers }>150\text{g}}{\text{Total tuber weight}})\times \;100$$

The grade of 150 grams represents the minimum marketable size standard for fresh potatoes in China, with tubers below this size being classified as substandard or suitable solely for starch processing.

### 2.5 Soil sampling and determination of soil physical and chemical properties

Soil samples from the 0–20 cm layer of each plot were collected both before potato sowing and at harvest. The five-point sampling method was employed, and the samples were mixed. Surface debris, such as plant residues and roots, was removed. The remaining portion of the soil samples were air-dried and passed through a 2 mm sieve for analysis of physical and chemical properties. Soil bulk density was determined by the stainless steel core method using 5.0 cm diameter × 5.0 cm height cores. Three replicate cores were taken from the 0–20 cm depth in each plot and dried at 105 °C for 24 h and weighed to calculate dry soil weight per unit volume and determine soil bulk density(BD) (Meng et al., 2019). The soil water-stable aggregates were measured using the wet sieving method (Li et al., 2007). 50 g of undisturbed soil after natural air-drying was weighed, soaked in distilled water for 5 min, and then transferred to the top layer of a set of sieves with pore sizes of 2 mm, 0.25 mm, and 0.053 mm from top to bottom. The vibrating sieve shaker was started, and wet sieving was carried out for 10 min at an amplitude of 3.8 cm and a frequency of 32 revolutions per minute. The soil aggregates on each sieve were washed into beakers with distilled water, and four particle size fractions of soil aggregates, namely >2 mm, 0.25–2 mm, 0.053 - 0.25 mm, and <0.053 mm, were separated. These aggregates were dried at 75 °C, cooled, and weighed for the determination of the percentage content of soil water-stable aggregates.

Formulas for calculating soil mean weight diameter (MWD) and geometric mean diameter (GMD):


$$MWD=\sum (x_{i}\times \frac{m_{i}}{m_{t}})$$

$$GMD=E_{XP}[\frac{\sum (mi\times \ln X_{i})}{\sum mi}]$$

Notation: MWD is the mean mass diameter (mm), GMD is the geometric mean diameter (mm); X_i_ is the average diameter of the i-th level aggregate (mm); m_i_ is the mass of the i-th level aggregate (g), and m_t_ is the total mass of the soil sample (g).

Soil pH was measured with a pH electrode (Leici, Shanghai, China) using a soil-to-water ratio of 1:2.5 (Wen et al., 2025). Soil organic carbon (SOC) content was assessed via the potassium dichromate volumetric method (Nie et al., 2024). Total nitrogen (TN) was determined by the Kjeldahl method (Chen et al., 2024b). The concentrations of NO_3_^--^N and NH_4_^+^-N in the soil were determined using a flow analyzer following extraction with KCl (Gong et al., 2019).

During the potato tuber expansion phase (after 60 days since the potato seedlings emerged), three potato plants with uniform growth and vigor were randomly selected from each treatment group. The plants were excavated to a depth of approximately 20 cm using a spade, maintaining a distance of 5 to 10 cm from the stem. After clearing impurities, the soil clinging to the root surface was brushed away. For each sample, 5 g of soil was collected and placed into a 10-ml centrifuge tube. The tubes were then numbered and stored in an incubator filled with dry ice. Three replicates were collected from each plot.

### 2.6 DNA extraction and high-throughput sequencing

DNA was extracted from the samples using the OMEGA Soil DNA Kit (D5635-02) (Omega Bio-Tek, Norcross, GA, USA). To prevent contamination from external bacteria and impurities, all procedures were conducted in a UV-sterilized clean bench, adhering to the kit’s instructions. The molecular size of the extracted DNA was assessed via 0.8% agarose gel electrophoresis. DNA quantification was conducted using a Nanodrop ND-2000 UV-Vis spectrophotometer (Thermo Fisher Scientific), and the extraction quality was verified with 1.2% agarose gel electrophoresis. For PCR amplification, 16S rRNA primers 338F (5’-ACTCCTACGGGAGGCAGCAG-3’) and 806R (5’-GGACTACHVGGGTWTCTAAT-3’) were used, while fungal amplification utilized ITS1F (5’-CTTGGTCATTTAGAGGAAGTAA-3’) and ITS2R (5’-GCTGCGTTCTTCATCGATGC-3’) (Klindworth et al., 2013). The PCR reaction began with pre-denaturation at 98 °C for 5 minutes. Each cycle included denaturing at 98 °C for 30 seconds, annealing at 53 °C for 30 seconds, and extending at 72 °C for 45 seconds, repeated for 25 cycles to enrich the DNA fragments. A final extension was performed at 72 °C for 5 minutes, and products were stored at 12 °C (Zhang et al., 2025a). Amplification results were analyzed using 2% agarose gel electrophoresis. Target fragments were excised and recovered using the Axygen gel recovery kit. The recovered PCR products were quantified using the Quant-iT Pico Green dsDNA Assay Kit and a Microplate reader (BioTek, FLx800).3 The sequencing library was prepared with the Tru Seq Nano DNA LT Library Prep Kit from Illumina, with index codes added. Before sequencing, the library’s quality was checked using an Agilent Bioanalyzer and the Agilent High Sensitivity DNA Kit (Han et al., 2025). Further quality evaluation was conducted with the Promega Quanti Fluor fluorescence quantification system using the Quant-iT Pico Green dsDNA Assay Kit. The library was then sequenced on the Illumina NovaSeq platform, generating paired-end reads of 200–450 bp.5.

### 2.7 Statistical analysis

Data organization was conducted using Excel 2021 and SPSS 27.0. SPSS 27.0 facilitated a one-way analysis of variance (ANOVA) and Tukey’s multiple comparisons (P < 0.05) to examine soil properties across different treatments. Origin2024 software was employed to create column and stacked charts. For the bacterial 16S rRNA gene analysis, the Green genes database was utilized. Sequence clustering and ASV division at a 97% similarity level were performed using QIIME software.6 The community composition of samples was statistically analyzed at the phylum level, followed by variance analysis and multiple comparisons. The alpha diversity indices, including Chao1, Shannon, Simpson, and Pielou-e, were computed by the QIIME2 (Liu et al., 2021).

Non-metric multidimensional scaling (NMDS) simplifies data structures by performing dimensionality reduction on the sample distance matrix, thereby describing the distribution characteristics of samples at a specific distance scale (Zhang et al., 2023). Linear Discriminant Analysis Effect Size (LEfSe) was applied to discover biomarkers, identifying taxa with differential abundances across different treatments. Linear Discriminant Analysis (LDA) was used to estimate the relative contributions of these taxa to differences among the six treatment groups, with a threshold of LDA > 3.0. Finally, distance-based redundancy analysis (db-RDA) was used to evaluate the relationships between soil physicochemical properties and soil microbial diversity and structure (Wang et al., 2022). In order to analyze the relationship between potato yield, soil microbial community characteristics, and environmental factors, SPSS 27.0 was used to perform Pearson correlation analysis between yield components and soil physicochemical parameters, and the Mantel test in the vegan package in R was conducted to assess the correlation between microbial community differences (Bray-Curtis distance) and the environmental distance matrix.

## 3 Results

### 3.1 Effects of organic fertilizer substitution for nitrogen fertilizer on potato yield

**Table 2** shows that substituting nitrogen fertilizer with organic fertilizer significantly impacted potato yield. In both experimental years, the T2 treatment produced markedly higher yields than the other treatments, with increases of 10.26% and 8.23% compared to the T0 treatment, respectively. Additionally, the T2 treatment exhibited the highest commercial potato rate and potato weight per plant. In 2024, the commercial potato rate for T2 rose by 12.24% relative to T0, although no significant difference was noted in potato weight per plant. In 2025, both the commercial potato rate and potato weight per plant for T2 were significantly greater than those for T0, with increases of 8.58% and 12%, respectively. These findings suggest that an optimal balance of organic and nitrogen fertilizers is crucial, as both excessive and insufficient organic fertilizer can reduce potato yield. Specifically, replacing 60% of chemical fertilizers with organic alternatives at equivalent nitrogen levels can increase yield while maintaining a relatively high commercial potato rate.

**Table 2:** Effects of different fertilization treatments on potato yield, commercial tuber rate, and tuber traits in 2024 and 2025 growing seasons.

Year	Treatment	Yield (t/hm^2^)	Commodity rate (%)	Tuber number per plant	Tuber weight per plant (g/plant)
2024	CK	40.09 ± 1.20d	59.94 ± 2.50d	5.06 ± 0.30a	956.10 ± 108.52c
	T0	55.02 ± 1.41b	70.75 ± 2.13b	5.68 ± 0.24a	1370.92 ± 59.48ab
	T1	50.31 ± 1.94c	69.53 ± 0.64b	4.64 ± 0.19a	1343.23 ± 38.85ab
	T2	60.67 ± 2.11a	79.41 ± 2.22a	5.30 ± 0.10a	1456.99 ± 20.82a
	T3	48.88 ± 1.06c	70.58 ± 0.97b	4.99 ± 0.50a	1162.05 ± 111.18bc
	N0	46.04 ± 0.72c	63.74 ± 4.05bc	5.16 ± 0.43a	1134.41 ± 116.27bc
2025	CK	35.58 ± 1.46d	38.79 ± 1.80d	4.93 ± 0.31a	830.83 ± 54.61d
	T0	48.25 ± 1.31b	65.76 ± 2.45b	4.69 ± 0.25a	1053.22 ± 14.46b
	T1	47.77 ± 1.03b	65.21 ± 1.79b	4.43 ± 0.08a	983.89 ± 18.49bc
	T2	52.22 ± 1.49a	71.40 ± 0.74a	5.07 ± 0.27a	1179.57 ± 33.55a
	T3	41.56 ± 0.49c	59.11 ± 2.41c	5.05 ± 0.18a	909.98 ± 35.84cd
	N0	37.95 ± 1.56cd	53.89 ± 0.97c	4.94 ± 0.31a	884.72 ± 49.51cd

Data are expressed as mean ± standard error (n = 4). Means within a column followed by different lowercase letters differ significantly (p < 0.05) by Duncan’s multiple range test after ANOVA.

### 3.2 Impact of replacing nitrogen fertilizer with organic fertilizer on soil physical and chemical properties

This section explores the effects of substituting nitrogen fertilizer with organic fertilizer on the physical and chemical characteristics of soil. The soil physicochemical properties presented in **Table 3** represent measurements taken at harvest in the 2025 growing season (second year), as this reflects the cumulative effects of the two-year fertilization treatments. **Table 3** highlights significant differences in the soil’s basic physical and chemical properties under various fertilization treatments. Soil bulk density ranged from 1.42 to 1.49 g/cm3³. Compared to the T0 treatment, the T2 and T3 treatments reduced soil bulk density by 2.7% and 2%, respectively, suggesting that organic fertilizer can lower soil bulk density. The SOC and TN levels in the T2 and T3 treatments were notably higher than those in the T0 and CK treatments. Specifically, the SOC content in the T2 and T3 treatments increased by 34.37% and 37.7%, respectively, while the TN content rose by 16.24% and 17.09% compared to T0. Additionally, NH_4_^_+_^-N and NO_3-_-N contents were significantly higher in the T0, T1, and T2 treatments than in others. The pH levels in the T2 and T3 treatments increased significantly, with no notable difference between the T0 and CK treatments. Analysis of **Table 4** reveals that the aggregates in the 0.25–2 mm particle size range are the dominant particle size class, accounting for 53.80%–57.60%. The content of 0.25–2 mm aggregates is the highest in the T2 treatment, significantly higher than that in the CK, T3, and N0 treatments. The content of 0.053–0.25 mm aggregates is the highest in the N0 and T3 treatments, significantly higher than that in the T0, T1, and T2 treatments, with the T2 treatment having the lowest content. Overall, the application of organic fertilizer significantly increases the content of large aggregates in the 0.25–2 mm particle size range and reduces the content of micro - aggregates in the 0.053–0.25 mm and <0.053 mm particle size ranges, which is conducive to promoting the formation of large soil aggregates. As shown in **Table 5**, different fertilization treatments in the 0–20 cm soil layer have a significant impact on the MWD and GMD values of soil water - stable aggregates. The MWD and GMD values of soil water - stable aggregates in the T2 and T3 treatments are significantly higher than those in the CK and T0 treatments. The MWD values are 7.27% higher than that in the CK treatment and 3.51% higher than that in the T0 treatment respectively; the GMD values are 16.88% higher than that in the CK treatment and 7.14% higher than that in the T0 treatment respectively. The CK treatment has the lowest MWD and GMD values, significantly lower than those of other treatments. Overall, the soil water - stable aggregates in the T2 and T3 treatments have the best stability, while the CK treatment has the worst stability, indicating that the application of organic fertilizer significantly improves the stability of surface soil aggregates.

**Table 3:** Effects of different fertilization treatments on soil physical and chemical properties.

Treatment	BD (g/cm^3^)	pH	SOC (g/kg)	TN (g/kg)	NH_4_^+^-N (mg/kg)	NO_3_N (mg/kg)	C:N
CK	1.47 ± 0.01a	7.76 ± 0.07b	12.64 ± 0.33c	0.87 ± 0.05c	2.36 ± 0.27c	10.86 ± 0.96bc	12.80 ± 0.83a
T0	1.47 ± 0.00a	7.75 ± 0.09b	12.57 ± 0.23c	1.17 ± 0.02b	3.96 ± 0.27a	14.00 ± 0.88ab	10.78 ± 0.02ab
T1	1.45 ± 0.00ab	7.92 ± 0.11ab	14.03 ± 0.54b	1.28 ± 0.03ab	3.45 ± 0.23ab	13.44 ± 0.39ab	9.98 ± 0.54c
T2	1.43 ± 0.01b	8.12 ± 0.05a	16.89 ± 0.26a	1.36 ± 0.02a	4.21 ± 0.37a	15.75 ± 0.46a	12.55 ± 1.34ab
T3	1.44 ± 0.00b	8.11 ± 0.04a	17.31 ± 0.26a	1.37 ± 0.03a	3.03 ± 0.08bc	11.70 ± 0.66bc	13.04 ± 0.85a
N0	1.46 ± 0.02ab	7.89 ± 0.05ab	12.51 ± 0.16c	0.97 ± 0.05c	2.26 ± 0.10c	10.06 ± 1.81c	12.90 ± 0.69a

The data are presented expressed as mean ± standard error. Different Significant letters indicate significant differences among the six fertilization treatments are indicated by different letters (p < 0.05).

**Table 4:** Soil water-stable aggregate size distribution (0–20 cm soil layer) under different fertilization treatments.

Soil depth (cm)	Treatment	≥2mm (%)	0.25–2 mm (%)	0.053-0.25mm (%)	<0.053 mm (%)
0-20	CK	24.95 ± 0.14a	54.71 ± 0.39c	17.83 ± 0.28ab	2.23 ± 0.08ab
	T0	25.66 ± 0.33a	56.15 ± 0.71b	16.34 ± 0.79bc	1.56 ± 0.11b
	T1	25.48 ± 0.29a	56.86 ± 0.30ab	15.63 ± 0.44c	1.62 ± 0.09b
	T2	24.87 ± 0.54a	57.60 ± 0.28a	15.05 ± 0.96c	1.68 ± 0.29b
	T3	23.32 ± 0.25b	54.58 ± 0.27c	19.00 ± 0.27a	2.26 ± 0.75ab
	N0	23.34 ± 0.03b	53.80 ± 0.92c	19.33 ± 0.45a	3.26 ± 0.55a

Data are expressed as mean ± standard error (n = 3). Means within a column followed by different lowercase letters differ significantly (p < 0.05) by Duncan’s multiple range test after ANOVA.

**Table 5:** Mean weight diameter (MWD) and geometric mean diameter (GMD) of soil water-stable aggregates as affected by fertilization regimes.

Soil depth(cm)	Treatment	MWD (mm)	GMD (mm)
0-20	CK	1.10 ± 0.01d	0.77 ± 0.01d
	T0	1.14 ± 0.00bc	0.84 ± 0.01bc
	T1	1.17 ± 0.01ab	0.89 ± 0.02ab
	T2	1.18 ± 0.01a	0.90 ± 0.01a
	T3	1.18 ± 0.02a	0.90 ± 0.03a
	N0	1.12 ± 0.01cd	0.81 ± 0.02cd

Data are expressed as mean ± standard error (n = 3). Means within a column followed by different lowercase letters differ significantly (p < 0.05) by Duncan’s multiple range test after ANOVA.

### 3.3 Composition of soil bacterial and fungal communities

**Figure 2:**
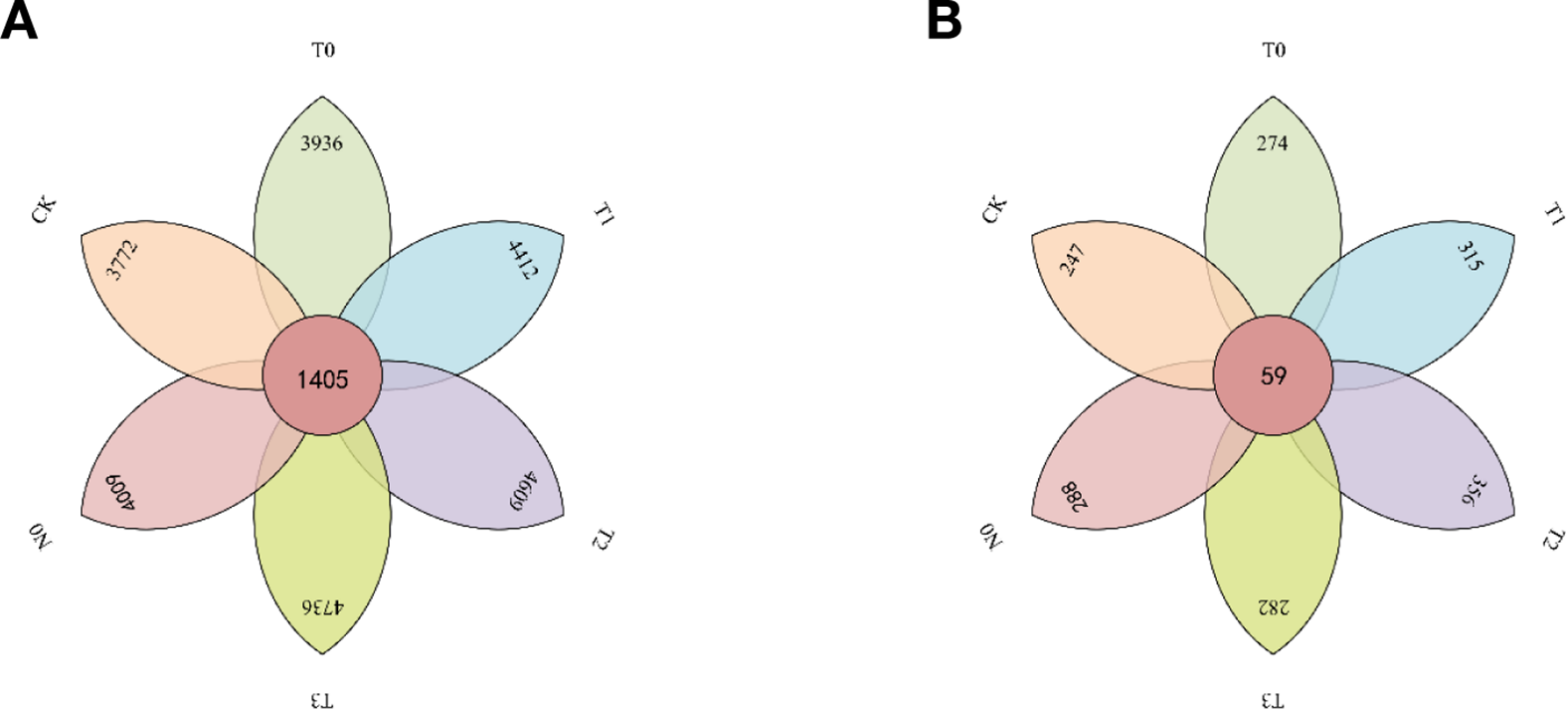
Venn diagrams showing the shared and unique amplicon sequence variants (ASVs) of soil bacteria **(A)** and fungi **(B)** across six fertilization treatments.

In this study, we utilized high-throughput sequencing technology to analyze how different fertilization treatments affect the rhizosphere soil microbial community in potatoes. **Figure 2A** presents the bacterial sequencing results, identifying a total of 26,879 Amplicon Sequence Variants (ASVs) in the rhizosphere soil. Among the treatments—CK, T0, T1, T2, T3, and N0—there were 1,405 common bacterial ASVs. The unique bacterial ASVs numbered 3,772, 3,936, 4,412, 4,609, 4,736, and 4,009, respectively. **Figure 2B** illustrates that the total number of ASVs in the fungal samples across all fertilization treatments was 1,821, with 59 common fungal ASVs across treatments. The unique ASVs for CK, T0, T1, T2, T3, and N0 were 247, 274, 315, 365, 282, and 288, respectively.

### 3.4 Alpha-diversity of bacteria and fungi under different fertilization treatments

**Figure 3:**
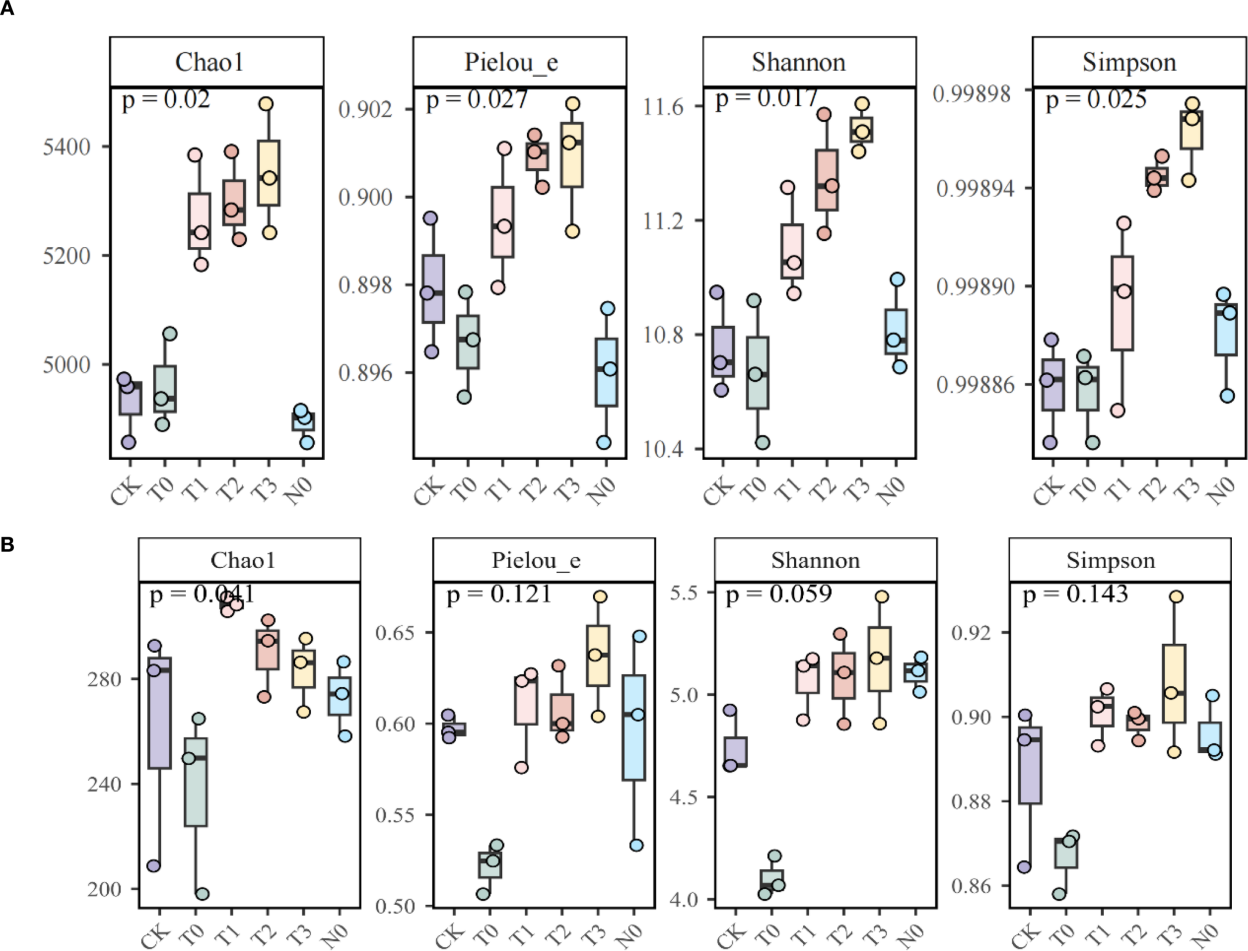
Alpha diversity indices (Chao1, Shannon, Simpson, Pielou_e) of rhizosphere soil bacterial **(A)** and fungal **(B)** communities under different fertilization treatments. There is a significant difference between treatments when p < 0.05. The values in the box plot are expressed as mean ± SD.

The alpha diversity of bacterial and fungal communities across different fertilization treatments was assessed using the Shannon, Simpson, Chao1, and Pielou indices. **Figure 3A** illustrates that the impact of these fertilization treatments on bacterial diversity was significant (P < 0.05). The Chao1 index, representing bacterial richness, was notably higher in treatments T1, T2, and T3 compared to CK and T0 treatments, with increases of 6.23%, 6.86%, and 7.92%, respectively, over the T0 treatment. Regarding the Shannon index, the T3 treatment was significantly higher than all other treatments except T2, showing a 7.98% increase compared to the T0 treatment.

The study examined the α-diversity of fungi in the rhizosphere soil of potatoes when organic fertilizer replaced nitrogen fertilizer. As illustrated in **Figure 3B**, the Chao1 index generally decreased with a higher substitution ratio of organic fertilizer, yet it remained higher than in the T0 treatment. Specifically, compared to T0, the Chao1 index in the T1 and T2 treatments increased significantly by 29.83% and 22.04%, respectively. Although the Pielou_e, Shannon, and Simpson indices were higher in treatments with organic fertilizer substitution than in T0, these differences were not statistically significant (P > 0.05).

### 3.5 β-diversity of bacteria and fungi under different fertilization treatments

**Figure 4:**
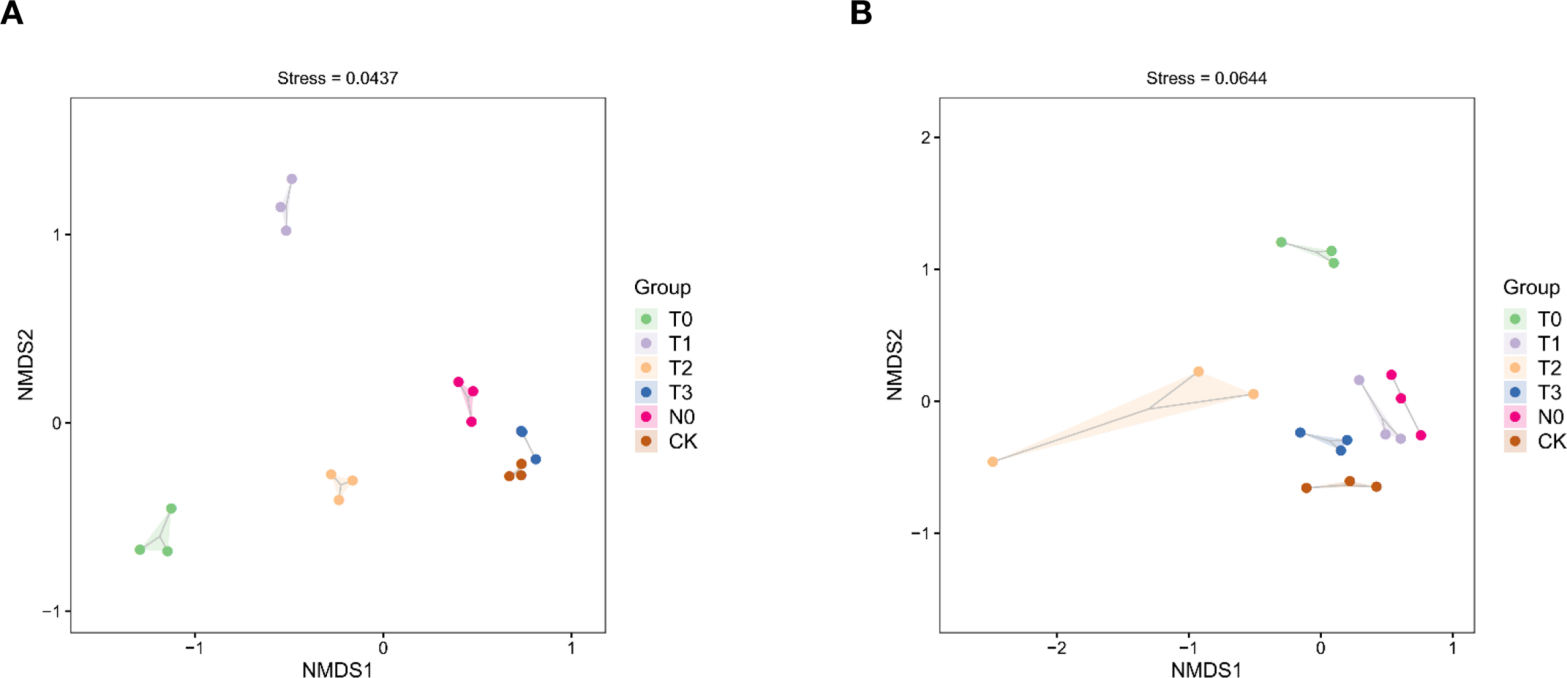
Non-metric multidimensional scaling (NMDS) analysis based on Bray-Curtis distance for soil bacterial **(A)** and fungal **(B)** community composition under six fertilization treatments. The stress values indicate reliable ordination results, and samples in the same treatment cluster closely with distinct separation among treatments. The treatments are as follows: CK represents no fertilization; T0 indicates 100% chemical fertilizer; T1 involves replacing 30% of nitrogen fertilizer with organic fertilizer; T2 involves replacing 60% of nitrogen fertilizer with organic fertilizer; T3 involves replacing 100% of nitrogen fertilizer with organic fertilizer; and N0 signifies no ap-plication of either nitrogen or organic fertilizer.

Further analysis of the β-diversity (NMDS) of the potato rhizosphere soil microbial community under various fertilization treatments was conducted. The non-metric multidimensional scaling (NMDS) plots for the bacterial and fungal communities (**Figure 4**) revealed distinct separations between these communities under different fertilization treatments. The bacterial community showed clear distribution differences among treatments along NMDS1 and NMDS2, with a stress value of 0.0437, indicating high reliability of the ordination results. Samples from different treatments clustered separately in the ordination space, highlighting significant differences in bacterial community structure across treatments. Notably, samples from treatments with relatively high organic fertilizer substitution ratios, such as T2 and T3, were closely grouped, suggesting similar bacterial community structures under these conditions. **Figure 4B** illustrates that the fungal communities under different fertilization treatments were distinctly separated along NMDS2, with samples from the same treatment clustering closely in the ordination space. This proximity indicates high similarity in fungal community structure within the same treatment. There was no overlap in the distribution areas of samples from different fertilization treatments, underscoring significant differences in fungal community structures among the treatments.

### 3.6 Composition of bacterial and fungal communities

**Figure 5:**
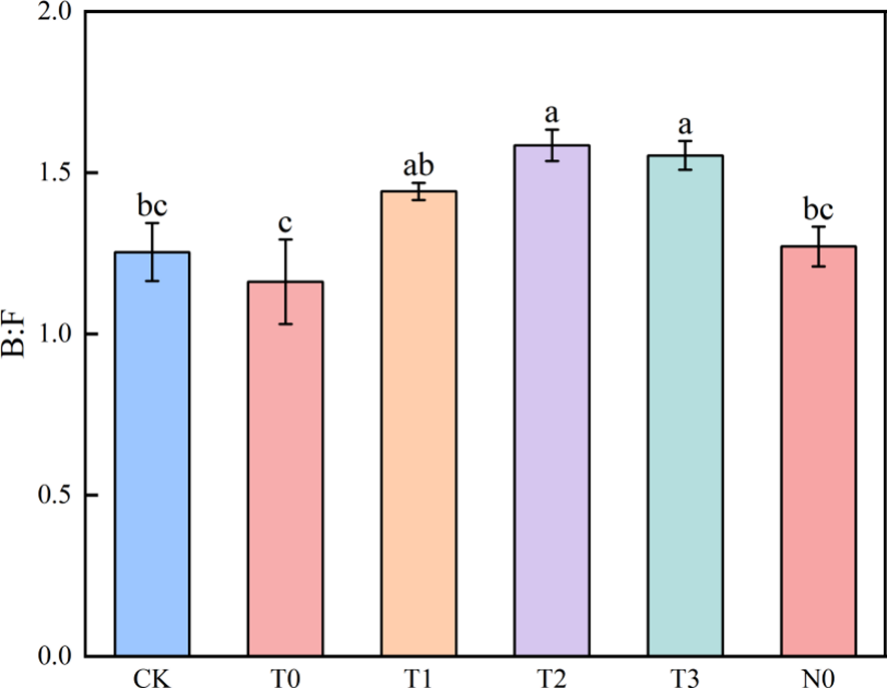
Ratio of soil fungi to bacteria abundance; different lowercase letters indicate significant differences among the four fertilization treatments (p < 0.05).

**Figure 5** shows the influence of different treatments on the ratio of soil microbial bacteria to fungi abundance (B:F). The ratio of soil bacteria to fungi abundance (B:F) exhibits significant differences among different treatments (P < 0.05). As can be seen from the figure, the B:F ratio of each treatment group ranges from 1.15 to 1.58, and overall, the bacterial abundance is higher than the fungal abundance. The B:F ratios of the T2 and T3 treatment groups are significantly higher than those of other treatments; the B:F ratio of the T0 treatment group is the lowest.

**Figure 6:**
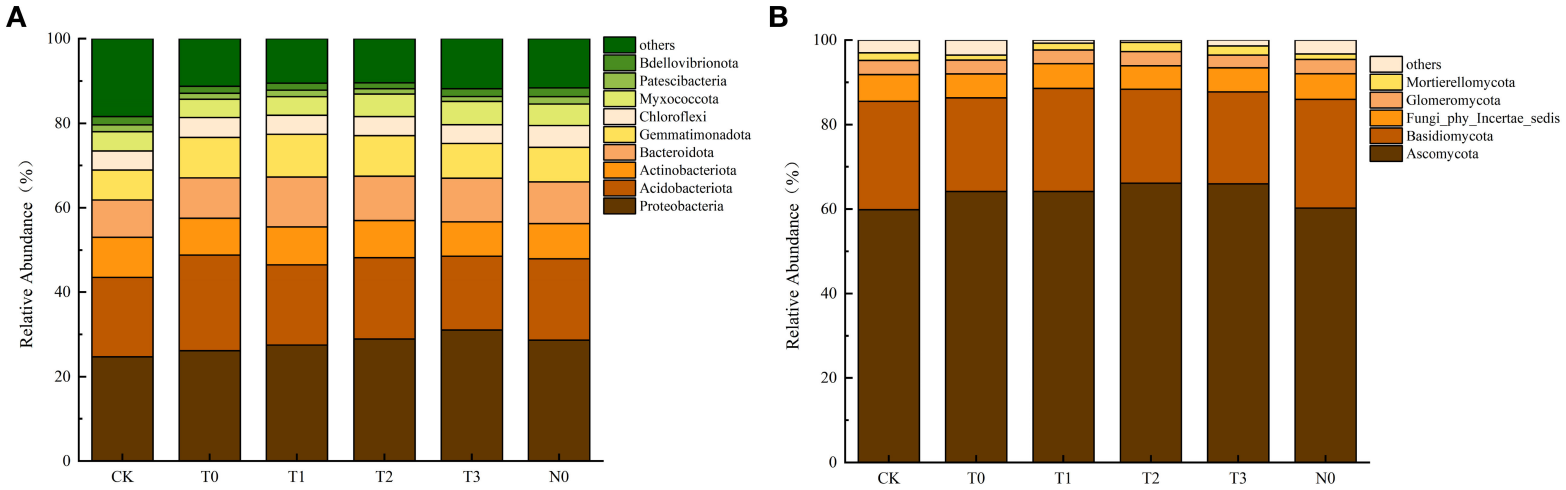
Relative abundance of dominant soil bacterial **(A)** and fungal **(B)** phyla under different fertilization treatments. The stacked column chart shows the composition and relative proportion of microbial phyla in each treatment.

All bacterial ASVs were classified into 34 phyla, 330 families, and 579 genera. **Figure 6A** shows that, at the phylum level, Proteobacteria exhibited the highest relative abundance within the soil bacterial community. Other phyla with notable abundances included Acidobacteria, Actinobacteria, Bacteroidetes, Gemmatimonadetes, and Chloroflexi. Treatment T0 significantly increased the relative abundance of Acidobacteria in the soil. In contrast, it significantly decreased the relative abundances of Proteobacteria and Bacteroidetes.

Six fungal phyla were identified in the soil samples. **Figure 6B** illustrates the relative abundance of these phyla. The dominant fungal phyla include Ascomycota, Basidiomycota, Fungi_phy_Incertae_sedis, Glomeromycota, and Mortierellomycota. Additionally, 5.64% of the fungal sequences could not be assigned to a specific phylum, indicating that a portion of the fungal community remains unidentified at the phylum level.

### 3.7 LEfSe analysis of bacterial and fungal communities

**Figure 7:**
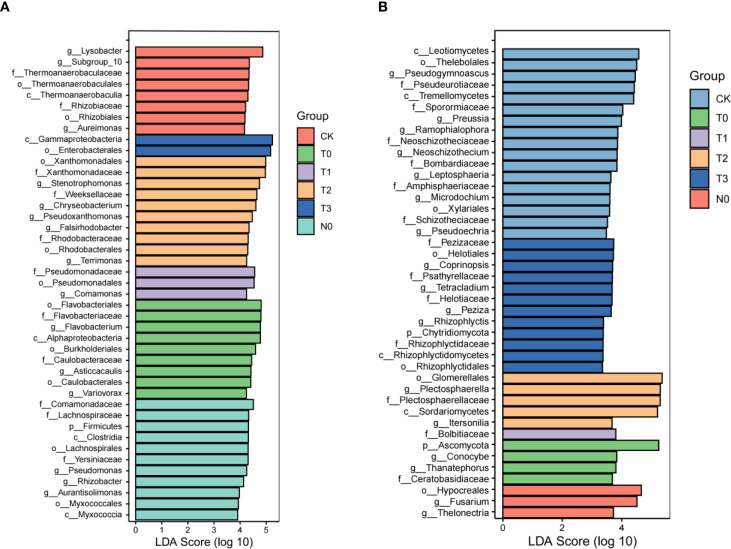
LEfSe analysis identifying biomarker taxa (LDA > 3.0) of soil bacteria **(A)** and fungi **(B)** responding to different fertilization treatments. The length of the bar represents the LDA score of differential biomarkers in each treatment.

The LEfSe analysis method systematically identified soil biomarker taxonomic groups across different fertilization treatments, with LDA scores greater than 3.0 and statistically significant differences (p < 0.05). This analysis highlighted the characteristic species responsible for differences between groups. As depicted in **Figure 7A**, there were 8 biomarkers for CK, 9 for T0, 3 for T1, 10 for T2, 2 for T3, and 11 for N0 treatments. Additionally, **Figure 7B** shows there were 17, 4, 1, 5, 12, and 3 fungal biomarkers for these treatments, respectively. Notably, compared to the CK treatment, fungal abundance decreased in the organic fertilizer substitution treatments.

In the six treated soils, bacterial biomarkers are mainly taxa linked to Proteobacteria, while fungal biomarkers are primarily associated with Ascomycota.

### 3.8 Relationships between soil microbial communities and environmental factors

**Figure 8:**
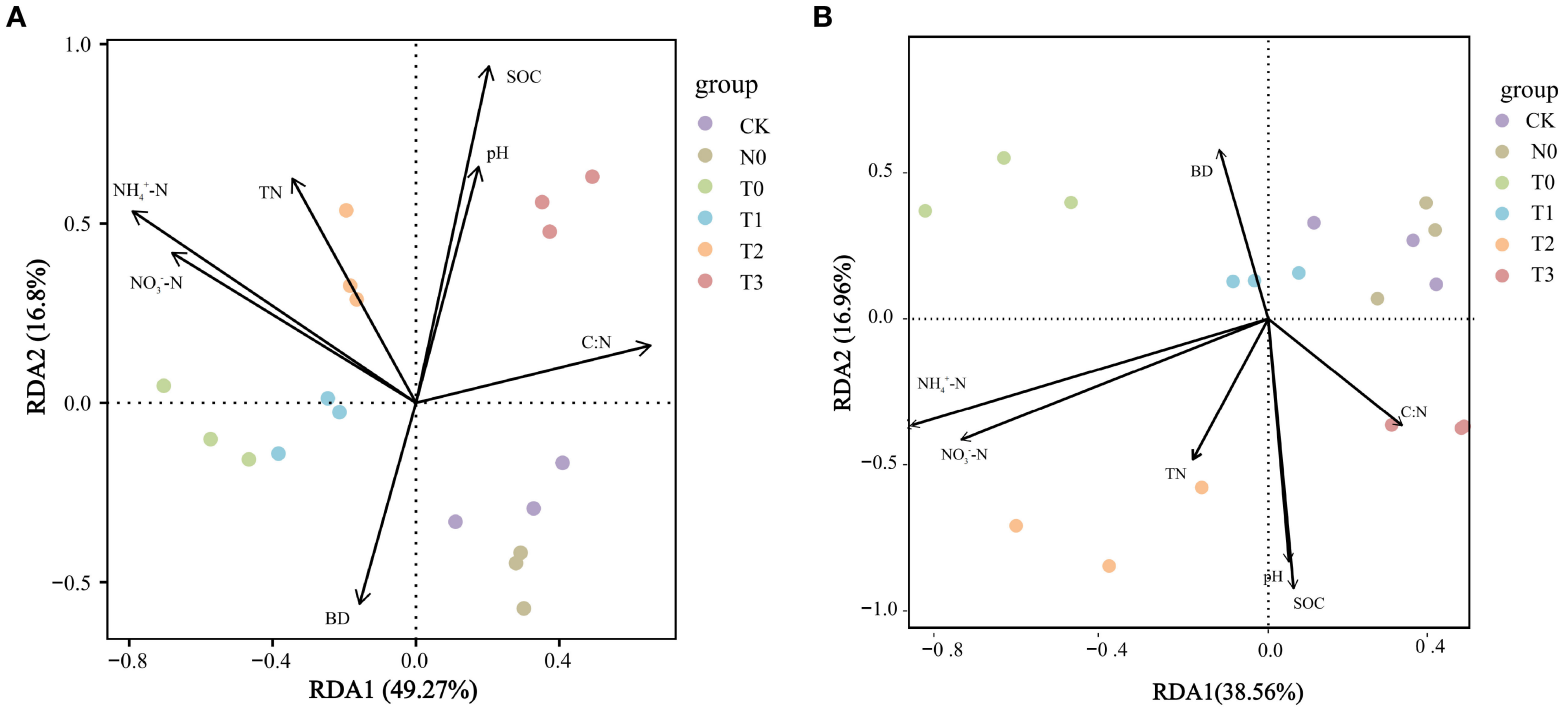
Distance-based redundancy analysis (db-RDA) illustrating the relationships between soil physicochemical properties and community structure of rhizosphere bacteria **(A)** and fungi **(B)** under different fertilization treatments.

**Figure 9:**
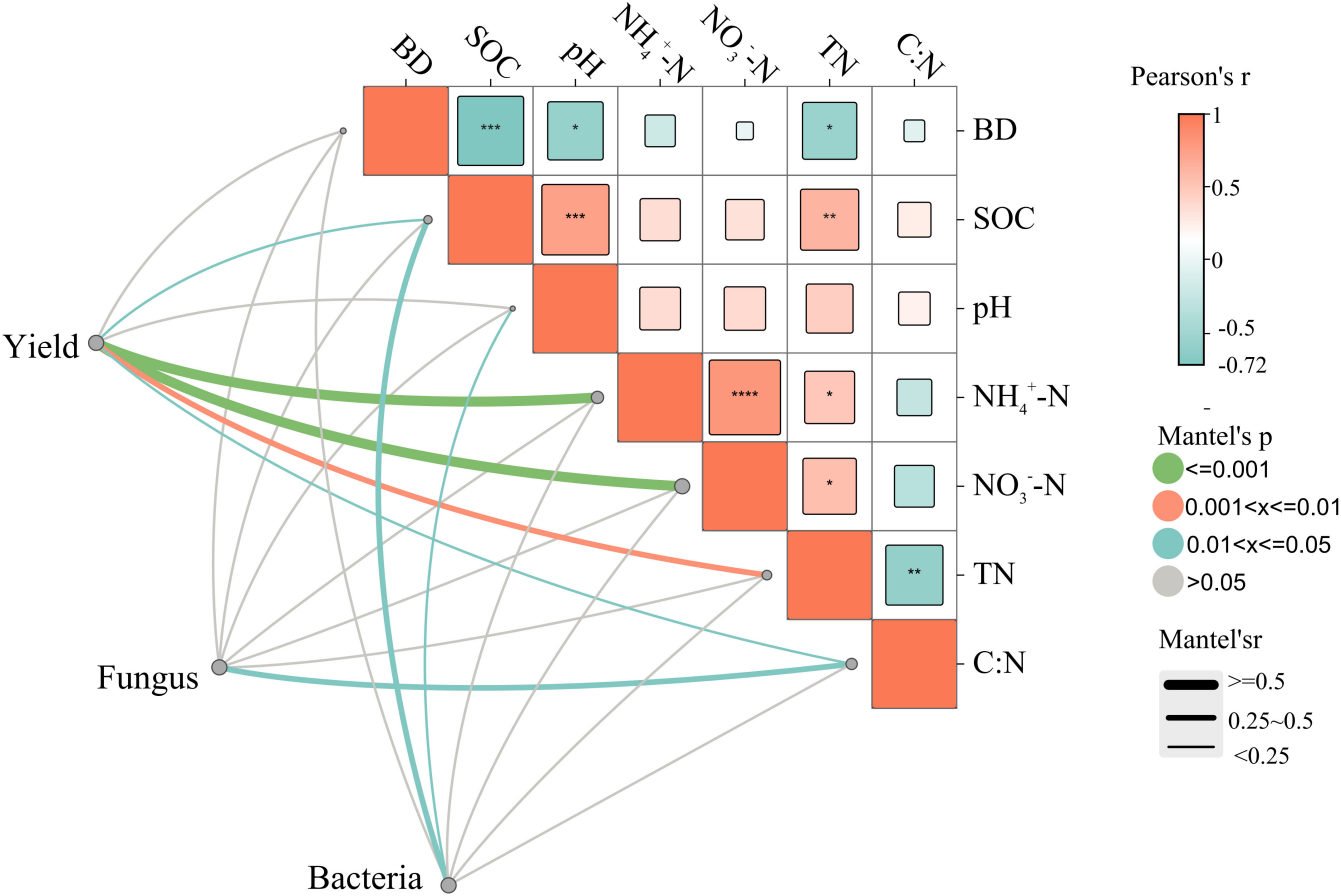
Mantel test illustrating the relationships among soil physicochemical properties, potato yield, and the alpha diversity of rhizosphere bacteria and fungi. Red lines represent positive correlations, while green lines denote negative correlations (*P < 0.05; **P < 0.01; ***P < 0.001).

Distance-based redundancy analysis (db-RDA) and Mantel test were combined to elucidate the relationships between soil physicochemical properties and microbial community structure/diversity. The db-RDA results (**Figure 8**) revealed that RDA2 accounted for 66.07% and 55.52% of the variance in bacterial and fungal communities, respectively. Soil SOC, NH_4+_-N, TN, and NO_3-_-N were identified as the key vectors driving both bacterial and fungal community differentiation (**Figure 8**). Specifically, the T2 bacterial community correlated positively with SOC, pH, TN, and C:N, while the T1 bacterial community correlated positively with NH_4+_-N, NO_3-_-N, and TN. Furthermore, the Mantel test (**Figure 9**) confirmed significant positive correlations between bacterial diversity and SOC, pH, and C:N (P < 0.05). Yield was significantly and positively correlated with NH_4+_-N and NO_3-_-N (P < 0.01)”.

## 4 Discussion

### 4.1 Impact of replacing nitrogen fertilizer with organic fertilizer on potato yield and soil properties

The decline in farmland soil quality in northern China’s arid regions is a major barrier to stable crop yields and sustainable agricultural development (Paramesh et al., 2023). This decline is characterized by low organic matter, poor structural stability, high bulk density, and reduced nutrient supply and efficiency. Although heavy reliance on chemical nitrogen fertilizers can temporarily sustain yields, it often leads to diminished soil ecological functions and increased environmental risks. Thus, developing a fertilization strategy that emphasizes organic matter input while reducing and optimizing chemical fertilizer use is crucial for improving soil quality in these arid regions. Soil fertility is crucial for achieving high crop yields and promoting sustainable agricultural development, as it directly affects crop growth and yield formation (Qi et al., 2025). Research consistently shows that partially replacing chemical fertilizers with organic fertilizers can effectively enhance soil fertility and boost crop yields (Adekiya et al., 2020; Anisuzzaman et al., 2021). Our study found that substituting 60% of nitrogen fertilizers with organic fertilizers significantly increased both the commercial potato rate and tuber weight per plant, thereby maximizing potato yield. This finding suggests that in the arid regions of Northwest China, replacing nitrogen fertilizers with organic fertilizers in the right proportion can optimize crop yield structure while maintaining nutrient supply.

However, when the proportion of organic fertilizers was increased beyond 60%, potato yields began to decline despite continued increases in soil microbial diversity. This indicates that enhanced microbial diversity does not always lead to higher crop yields. The likely reason is that at higher replacement levels, organic nitrogen remains in an organic form, requiring microbial mineralization before crops can absorb it (Chen et al., 2024a). This process can result in an insufficient nitrogen release rate during the potatoes’ rapid growth phase, failing to meet their high nitrogen demand (Shaji et al., 2021), thereby limiting yield formation. This observation aligns with the findings of Han X et al (Han et al., 2025). Therefore, improving yields depends not only on increasing soil nutrients and microbial populations but also on aligning the nutrient supply with crop demand.

Organic fertilizer substitution treatments generally increased soil total nitrogen and organic carbon levels. Notably, the high-substitution ratio treatment led to the most significant accumulation of soil organic carbon (SOC) and total nitrogen (TN), highlighting the advantages of organic fertilizers in enhancing soil carbon storage and nutrient pools (Han et al., 2021). However, solely using organic fertilizers or substituting them at a high ratio did not yield the best results, emphasizing the importance of balancing available and slow-release nutrients for optimal yields. Chemical nitrogen fertilizers quickly supply available nitrogen during the early growth stages, while organic fertilizers sustain nutrient availability in later stages through slow release and soil structure enhancement. This synergy is crucial for maintaining high potato yields and soil health (Udumann et al., 2025).

This study found that soil pH was lowest in treatments using only chemical fertilizers and highest when organic fertilizers replaced 60% of nitrogen fertilizers. This suggests that organic fertilizers significantly mitigate soil acidification from prolonged chemical fertilizer use. The presence of alkaline ions in organic fertilizers likely contributes to this effect by buffering the acidification that occurs during the nitrification of ammonium nitrogen (Zhou et al., 2013; Wu et al., 2020). Improved soil pH enhances nutrient availability and creates a favorable environment for a stable microbial community (Navarrete et al., 2015). Replacing chemical fertilizers with organic fertilizers in the right proportion can enhance soil fertility indicators, such as soil organic carbon (SOC) and total nitrogen (TN). This practice also aids in restoring soil structure and biological activity by lowering soil bulk density and improving soil pH and nutrient distribution. Consequently, it helps mitigate the decline in farmland soil quality in the arid regions of northern China. Compared to using only chemical fertilizers, the combined application of organic and inorganic fertilizers is more effective for achieving short-term yield increases and long-term soil fertility improvements. This approach offers a viable strategy for potato production in arid regions to maintain stable yields, improve quality, and conserve soil.

### 4.2 Differential effects of organic fertilizer substitution for nitrogen fertilizer on soil microbial diversity

Soil microbial diversity serves as a crucial indicator of the stability and functional potential of soil ecosystems. Studies consistently demonstrate that applying organic fertilizers generally boosts the α-diversity of soil bacteria and fungi (Zhang et al., 2021; Ma et al., 2023). This enhancement occurs because organic fertilizers supply microorganisms with abundant carbon sources and diverse nutrient substrates, fostering microbial growth and reproduction (Zhou et al., 2016). Our study found that the long-term exclusive use of chemical fertilizers significantly decreased the α-diversity of rhizosphere soil bacteria. In contrast, replacing nitrogen fertilizers with organic alternatives markedly increased bacterial diversity. These findings align with previous research in crop systems such as dryland wheat, apples, and cotton (Zhao et al., 2023; Song et al., 2024; Abudurezike et al., 2025).

In contrast, fungal α-diversity did not significantly respond to fertilization measures, aligning with Francioli D et al.’s findings (Francioli et al., 2016). The Chao1 index of fertilizer treatment was significantly lower than that of other treatments.This suggests that bacterial communities are more sensitive to fertilization changes, whereas fungal communities exhibit greater environmental tolerance. One possible explanation is that fungi possess higher functional redundancy in decomposing complex organic matter, making them less affected by short-term nutrient fluctuations (Samson et al., 2020). Conversely, bacteria rely more on readily available carbon sources and inorganic nutrients, leading to a quicker response to organic fertilizer inputs (Shen et al., 2023; Zhang et al., 2024). It may also be because organic fertilizers increase organic matter, provide sources of carbon and energy, change the competition and interactions among fungi, affect soil environmental factors, and thereby influence fungal diversity. These findings further indicate that replacing nitrogen fertilizer with organic fertilizer primarily influences soil ecological processes by regulating bacterial community diversity.

### 4.3 Impact of replacing nitrogen fertilizer with organic fertilizer on soil microbial community structure and its environmental drivers

The substitution of organic fertilizer for nitrogen fertilizer significantly influenced the soil microbial community structure. This change was evident in the increased diversity and abundance of beneficial microorganisms. The shift in microbial composition was primarily driven by alterations in soil pH, organic matter content, and nutrient availability, which are critical environmental factors (Li et al., 2021). Research has shown that organic fertilizers enhance soil health by improving its physical and chemical properties. These improvements create a more favorable environment for microbial activity, leading to a more balanced and resilient microbial community. The enhanced microbial diversity contributes to better nutrient cycling and soil fertility, which are essential for sustainable agricultural practices (Bertola et al., 2021). In experiments, soils treated with organic fertilizers demonstrated higher microbial biomass and activity compared to those treated with synthetic nitrogen fertilizers. This increase in microbial activity is attributed to the higher organic matter content provided by organic fertilizers, which serves as a food source for microorganisms. Overall, the transition from nitrogen to organic fertilizers offers a promising approach to improving soil health and sustainability. Future research should focus on optimizing the use of organic fertilizers to maximize their benefits on soil microbial communities and environmental health.

Variations in soil microbial composition can differ significantly depending on the types of organic fertilizers applied. This is because different organic fertilizers vary in terms of chemical properties, nutrient content, and organic matter stability, thereby exerting selective effects on soil microbial communities (Bai et al., 2024). Research indicates that the type of organic fertilizer is a key factor driving the variation in the β - diversity of soil bacteria and fungi, and its influence is often stronger than the application rate or frequency (Cui et al., 2023). For instance, in loess paddy soils, long - term application of chemical fertilizers can alter the soil microbial community structure, while the combined application of organic and inorganic fertilizers can increase crop yields by enhancing the complexity of the microbial community (Yang et al., 2024). An analysis of the effects of three types of fertilizers on the soil microbial structure reveals that the soil bacterial community structure is influenced by both the fertilization method and the planting season, whereas the fungal community is only affected by the fertilization method (Zhang et al., 2023). Therefore, the types of organic fertilizers significantly influence the structure and diversity of soil microbial communities through their unique chemical compositions, nutrient release patterns, and impacts on soil physical and chemical properties.

Fertilization methods significantly influenced the rhizosphere soil microbial community structure. In the bacterial community, Proteobacteria, Acidobacteria, and Actinobacteria consistently dominated, aligning with general observations in long-term fertilized farmlands (Ling et al., 2022). The exclusive use of chemical fertilizers significantly increased the relative abundance of Acidobacteria while reducing that of Proteobacteria and Bacteroidetes. This shift may be linked to the oligotrophication and acidification of the soil environment following chemical fertilizer application. Acidobacteria, being oligotrophic, thrive in low-organic-matter and acidic conditions (Song et al., 2022). Conversely, substituting chemical fertilizers with organic fertilizers significantly enriched Proteobacteria and Actinobacteria, which are associated with organic matter decomposition, nitrogen transformation, and plant growth-promoting functions (Wang et al., 2021). The treatment substituting 60% of nitrogen fertilizer with organic fertilizer exhibited the optimal community structure, with a higher number of ASVs and more biomarkers identified by LEfSe than other treatments. This finding suggests that this substitution ratio effectively supports microbial growth while maintaining the stability and functional diversity of the community structure. It provides direct evidence for the notion that “the appropriate substitution ratio is key to optimizing microbial functions rather than merely increasing their quantity”.

In the fungal community, Ascomycota and Basidiomycota consistently held dominant positions. The use of organic fertilizers did not change their core structure but adjusted their relative abundances and the composition of subordinate taxa. This stability may be attributed to fungi’s competitive advantage in utilizing complex organic substrates, allowing them to maintain a stable community structure under varying fertilization conditions. In contrast to bacteria, the fungal community exhibited less differentiation, suggesting that its response to fertilization interventions requires a longer time frame.

Environmental factor analysis revealed that SOC, TN, and inorganic nitrogen forms were key drivers of changes in bacterial community structure, while SOC and the C:N ratio significantly influenced the fungal community. This suggests that substituting organic fertilizers for nitrogen fertilizers indirectly shapes the microbial community structure by altering soil carbon and nitrogen status, thereby impacting the rhizosphere microecological environment and crop growth. Soil pH is regarded as one of the most significant factors influencing the structure of microbial communities (Philippot et al., 2023). In acidic soils, bacteria belonging to the Acidobacteria phylum tend to be more dominant, while in neutral to alkaline soils, Proteobacteria and Actinobacteria are more abundant. Long-term fertilization can alter the soil pH, thereby affecting the composition and function of microbial communities (Chen et al., 2020). The content of soil organic carbon (TOC) is closely related to the structure and function of microbial communities (Fu et al., 2024). Long-term greenhouse cultivation significantly alters the structure and diversity of soil microbial communities and affects carbon cycling-related functional genes, which is related to the dynamic changes of organic matter (Zhang et al., 2025b). Changes in soil microbial communities are usually the result of the combined action of multiple environmental factors.

## 5 Conclusions

Research indicates that in the arid region of Northwest China, substituting 60% of chemical nitrogen fertilizers with organic fertilizers significantly enhances soil physical and chemical properties, boosting potato yield. Compared to using only chemical fertilizers, this substitution reduces soil bulk density, promotes the formation of macroaggregates, and enhances the stability of water-stable aggregates as indicated by increased MWD and GMD values, and increases soil organic carbon, total nitrogen, and inorganic nitrogen levels. It also significantly enhances the diversity of rhizosphere soil bacteria, promoting the growth of Proteobacteria and Actinobacteria, while minimally affecting the fungal community structure. Key factors driving changes in the bacterial community include soil organic carbon, inorganic nitrogen, and pH. These findings suggest that replacing nitrogen fertilizers with organic alternatives in a balanced ratio supports high potato yields and improves soil quality by regulating the soil microbial community and nutrient supply.

## Data Availability

The original contributions presented in the study are publicly available. This data can be found here: NCBI Sequence Read Archive (SRA), BioProject accession PRJNA1477056, SRA accession SRP708483, accessible at https://www.ncbi.nlm.nih.gov/bioproject/PRJNA1477056.
